# FGF8-mediated TRIM16 regulation promotes K48-linked ubiquitination and degradation of RIG-I to facilitate Influenza a virus immune evasion

**DOI:** 10.1080/21505594.2026.2677346

**Published:** 2026-05-22

**Authors:** Ran Wei, Huixia Zhang, Kaihui Cheng, Song Wang, Zhen Yuan, Sisi Ma, Zhijun Yu

**Affiliations:** a Poultry Institute, Shandong Academy of Agricultural Sciences, Ji’nan, China; b Shandong Provincial Key Laboratory of Livestock and Poultry Breeding (PKL2024B15), Ji’nan, Shandong, China; c Institute of Animal Science and Veterinary Medicine, Shandong Academy of Agricultural Sciences, Ji’nan, China; d Department of Clinical Laboratory, Shandong Provincial Hospital Affiliated to Shandong First Medical University, Ji’nan, Shandong, China; e School of Biological Science and Technology, University of Jinan, Ji’nan, China; f School of Animal Science and Technology, Ningxia University, Lan’zhou, China

**Keywords:** Influenza A virus, H13N2, H1N1, FGF8, TRIM16, RIG-I

## Abstract

Influenza A virus (IAV) exhibits notable genetic diversity and cross-species transmission capacity, posing a continuous challenge to public health. Elucidating host immune regulatory mechanisms is crucial for identifying new antiviral targets that can overcome viral resistance. Here, using human A549 lung epithelial cells as the primary model, we identify fibroblast growth factor 8 (FGF8) as a crucial host factor whose expression is significantly elevated during infection by various IAV subtypes (including H1N1, H13N2, H9N2, and PR8). Through gain- and loss-of-function assays, we demonstrate that FGF8 specifically enhances viral replication at the post-entry stage by suppressing interferon-beta (IFN-β) and interferon-stimulated genes (ISGs) expression. Mechanistically, FGF8 reduces retinoic acid-inducible gene I (RIG-I) protein stability via K48-linked polyubiquitination without affecting its mRNA levels. Ubiquitination identifies Lysine 258 (K258) on RIG-I as the essential modification site; notably, a K258R mutation prevents RIG-I degradation and restores IFN-β induction. Furthermore, TurboID-based proximity labeling captures the close spatial association of FGF8 with both tripartite motif containing 16 (TRIM16) and RIG-I, revealing that FGF8 acts as a molecular scaffold to recruit the E3 ligase TRIM16 to RIG-I. Consistently, TRIM16 silencing replicates the antiviral effects of FGF8 knockdown. Collectively, our findings demonstrate that FGF8 recruits TRIM16 to degrade RIG-I, thereby facilitating viral immune evasion. Disrupting this interaction offers a potential avenue for anti-influenza A virus intervention.

## GRAPHICAL ABSTRACT

**Figure:**
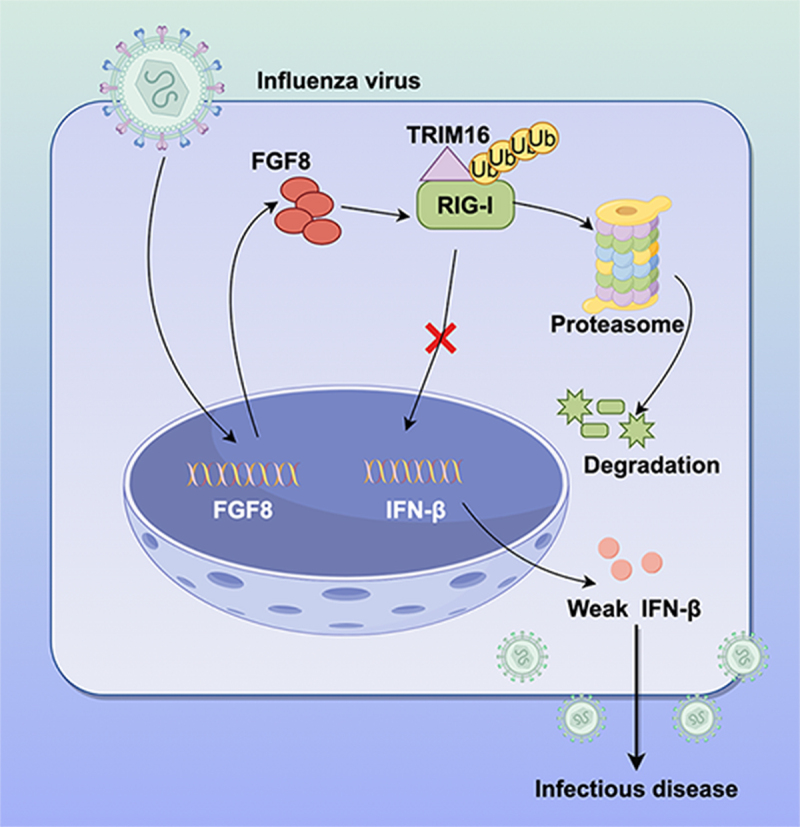
The diagram illustrates that FGF8 inhibits the antiviral IFN-I response through negative regulation of RIG-I.

## Introduction

Influenza viruses are among the most significant pathogens causing respiratory infections worldwide, characterized by high transmissibility and variability. Influenza A virus, a predominant influenza type, is widespread in nature and infects diverse hosts, including humans, birds, swine, horses, and marine mammals. A novel H1N1 subtype swine influenza virus, prevalent in swine herds, has been isolated in Shandong Province, China. This strain is a new influenza A virus formed by reassortment between the 2009 pandemic H1N1 virus and swine influenza viruses [1]. The H13N2 avian influenza virus primarily circulates among waterfowl, and its cross-species transmission risk makes it a potential public health concern [2–6]. Investigating the interplay between host factors and viral replication is crucial for understanding viral pathogenesis and developing novel antiviral strategies.

Fibroblast growth factor 8 (FGF8), an essential FGF family member, plays a vital role in embryonic development, tissue repair, cell proliferation, differentiation, and tumorigenesis [7–10]. FGF8 binds to FGFR, triggering downstream pathways such as Ras/MAPK, PI3K/Akt, and JAK/STAT, which control diverse cellular processes [11,12]. FGF8’s involvement in tumorigenesis has garnered significant interest recently, yet its precise role during viral infections is still not well understood [13–15]. The IFN-β signaling pathway plays a crucial role in defending the host against viral infections. Many viruses, including influenza, have evolved strategies to avoid host immune detection and suppress interferon signaling activation. Retinoic acid-inducible gene I (RIG-I) is essential for detecting viral RNA and plays a key role in regulating antiviral immune [16].

In this study, we identified FGF8 as a novel broad-spectrum proviral host factor. Initially identified via transcriptomic analysis of cells infected with swine H1N1 and avian H13N2 viruses, we found that FGF8 expression is consistently upregulated by diverse IAV subtypes (including H1N1, H13N2, H9N2, and PR8). We demonstrate that FGF8 suppresses the type I IFN response and enhances the replication of these these IAV strains. Mechanistically, FGF8 acts as a molecular scaffold to recruit the E3 ubiquitin ligase tripartite motif containing 16 (TRIM16) to RIG-I, facilitating the K48-linked polyubiquitination and subsequent proteasomal degradation of RIG-I. It identifies potential therapeutic targets for antiviral strategy development.

## Materials and methods

### Cells and viruses

Human lung adenocarcinoma cell line A549 (Procell Life Science & Technology Co., Ltd., Wuhan, China; Cat. No. CL-0016), human embryonic kidney cell line HEK293T (Procell Life Science & Technology Co., Ltd., Wuhan, China; Cat. No. CL-0005), and Madin-Darby canine kidney cell line MDCK (Procell Life Science & Technology Co., Ltd., Wuhan, China; Cat. No. CL-0154) were used for virus infection experiments, protein interaction validation experiments, and virus titration assays, respectively. A549 cells were cultured in F12K medium (Biyuntian Biotechnology Co., Ltd.) supplemented with 10% fetal bovine serum (FBS, BI) and 1% penicillin-streptomycin (P/S, Gibco). HEK293T and MDCK cells were maintained in DMEM medium (Biyuntian Biotechnology Co., Ltd., Shanghai, China) with 10% FBS and 1% P/S. All cell lines were maintained at 37°C in a humidified incubator with 5% CO_2_. The laboratory preserved the following influenza A virus strains: H1N1 (A/swine/China/Qingdao/2018), H13N2 (A/black-tailed gull/Weihai/115/2016), H9N2 (A/Chicken/Shandong/6/1996), and PR8 (A/Puerto Rico/8/1934). The vesicular stomatitis virus encoding green fluorescent protein (VSV-GFP) was kindly provided by Song Wang Ph.D. (Department of Clinical Laboratory, Shandong Provincial Hospital affiliated to Shandong First Medical University).

### RNA-sequencing

RNA was extracted from A549 cells 24 hours post-infection with H1N1 or H13N2, with uninfected A549 cells serving as the negative control. Each group included three biological replicates. The samples were sent to HuaDa BGI for RNA-seq analysis. BGI was responsible for constructing the mRNA library and performing transcriptome data analysis. For detailed experimental procedures, please refer to BGI’s official website. RNA-seq data were quantified using Fragments Per Kilobase of transcript per Million mapped reads (FPKM).

### RT-qPCR

Total RNA was isolated with the SteadyPure RNA Extraction Kit (AG21024, AGbio, Changsha, China). The quantitative reverse transcription PCR (RT-qPCR) was performed using the Evo M-MLV One-Step RT-qPCR Kit (AG11732, AGbio, Changsha, China). GAPDH was used as internal control. Relative expression levels were calculated using the 2^−ΔΔCt^ method. The primer sequences used for RT-qPCR are listed in Table S1.

### Western blot assay

Total protein was extracted from cells using RIPA lysis buffer (medium strength) supplemented with PMSF and phosphatase inhibitors (Biyuntian Biotechnology Co., Ltd., Shanghai, China). Proteins (30 μg per lane) underwent SDS-PAGE separation, PVDF transfer, and blocking with 5% milk in TBST. They were incubated with primary antibodies overnight at 4°C, followed by secondary antibodies for 1 hour at room temperature, and detected using SuperPico ECL. Refer to Table 1 for the list of antibodies.

**Table 1.** Antibodies used in Western blot.

Antibody name	Supplier	Catalog number	Dilution
**Primary Antibodies**			
Influenza A virus NP	GeneTex	GTX636199	1:2000
Influenza A virus PB1	GeneTex	GTX125923	1:2000
Influenza A virus PB2	GeneTex	GTX125926	1:2000
Rig-I	CST	3743T	1:1000
TBK1/NAK	CST	3504T	1:1000
Phospho-TBK1/NAK	CST	5483T	1:1000
IRF-3	CST	4302T	1:1000
Phospho-IRF-3	CST	79945T	1:1000
β-Actin	CST	4967S	1:1000
Anti-FGF8 antibody	Abcam	ab89550	1:500
TRIM16 Polyclonal antibody	Proteintech	24,403–1-AP	1:1000
HA tag Polyclonal antibody	Proteintech	51,064–2-AP	1:1000
FLAG tag Polyclonal antibody	Proteintech	20,543–1-AP	1:1000
MYC tag Monoclonal antibody	Proteintech	60,003–2-Ig	1:1000
VSV G-tag Ab	Abways	AB0053	1:2000
**Secondary Antibodies**			
HRP-Goat Anti-Rabbit	Proteintech	SA00001-2	1:5000
HRP-Goat Anti-Mouse	Proteintech	SA00001-1	1:5000

### TCID50

MDCK cells were seeded into 96-well cell culture plates and cultured until reaching 90% confluence. Subsequently, the samples were subjected to 10-fold serial dilutions (from 10^−1^ to 10^−8^), and the diluted samples were inoculated into the 96-well plates, with 8 replicate wells for each dilution. Uninfected wells were set as negative controls. After inoculation, the plates were incubated at 37°C with 5% CO_2_ for 4 days. For H1N1 virus, cytopathic effects (CPE) were observed under a microscope, while for H13N2 virus, the expression of viral nucleoprotein (NP) protein was detected using immunofluorescence. Finally, the 50% tissue culture infectious dose (TCID50) value was calculated using the Reed-Muench method to evaluate the infectivity of the virus.

### ELISA assay

The collected samples were analyzed using a human β-interferon (IFN-β) ELISA kit (mlbio, Cat. No. ml029388, Shanghai, China) according to the manufacturer’s instructions.

### Construction of A549 cell lines with stable silencing of FGF8/TRIM16

FGF8- and TRIM16-targeting short hairpin RNAs (shRNAs) were designed with the BLOCK-iT RNAi Designer (Thermo Fisher Scientific), synthesized by TsingKe (Beijing, China), and inserted into pLKO.1. Lentiviral particles were produced by co-transfecting HEK293T cells with pLKO.1-shRNA, psPAX2, and pMD2.G constructs. In brief, HEK293T cells were seeded into 100-mm cell culture dishes and cultured until the cell confluence reached 70%-80%. Subsequently, Lipofectamine 2000 was used to co-transfect 8 μg of pLKO.1-shRNA, 8 μg of psPAX2, and 4 μg of pMD2.G into the HEK293T cells. After transfection, the cell culture supernatant was collected at 48 hours post-transfection. The collected supernatant was centrifuged at 8000 rpm for 5 minutes to remove cell debris, yielding clarified viral supernatant. Polybrene was then added to the viral supernatant at a final concentration of 5 μg/mL to enhance viral infection efficiency. The prepared viral supernatant was subsequently used to infect target A549 cells. After 72 hours of incubation post-infection, 1 μg/mL puromycin was added to the culture medium to select cells successfully transduced with the lentiviral particles. The selection process lasted for 5 days, ensuring the establishment of a stable cell line for further experiments. The effectiveness of the knockdown was validated using RT-qPCR and immunoblotting. shRNA sequences are listed in Table S2.

### Dual-luciferase reporter assay

The dual-luciferase reporter assay was performed using HEK293T cells as the model. The experiment was conducted in 24-well plates, with approximately 5 × 10^4^ cells seeded per well. Cells were cultured in DMEM medium supplemented with 10% FBS at 37°C in a humidified atmosphere containing 5% CO_2_ until they reached 70%-80% confluence. Subsequently, the firefly luciferase reporter plasmid (firefly reporter construct), Renilla luciferase control plasmid (pRL-TK, used as an internal control), and other experimental plasmids were co-transfected into the cells according to the manufacturer’s instructions for Lipofectamine 2000. After 4–6 hours of incubation post-transfection, the medium was replaced with fresh DMEM, and the cells were cultured for an additional 24 hours. After completing the transfection, the medium was discarded, and 100 μL of cell lysis buffer was added to each well. The lysates were incubated at room temperature for 15 minutes to ensure complete cell lysis. The lysates were then collected, and the luminescence signals of firefly luciferase and Renilla luciferase were measured using a luminometer, following the instructions provided in the dual-luciferase assay kit (Yeasen, 11402ES60). To ensure data accuracy, the firefly luciferase activity was normalized to Renilla luciferase activity by calculating the ratio of firefly signal to Renilla signal, yielding the normalized firefly luciferase activity. The final experimental data were recorded for analysis.

### Immunofluorescence assay

A549 cells cultured on glass coverslips were infected with H13N2 for 24 hours, then fixed with 4% paraformaldehyde for 20 minutes, permeabilized using 0.1% Triton X-100 for 20 minutes and blocked with 5% BSA for 1 hour at room temperature. Cells were incubated overnight at 4°C with anti-TRIM16 (Proteintech, 24,403–1-AP, 1:50) and anti-RIG-I/DDX58 (Proteintech, 67,556–1-Ig, 1:200), followed by incubation with Alexa Fluor 488 goat anti-rabbit and Alexa Fluor 594 goat anti-mouse secondary antibodies (Abways, AB0141/AB0152, 1:500, 1 h, RT). Hoechst was used for nuclear counterstaining, and images were captured via confocal microscopy. PBS washes were performed between steps.

### Co-immunoprecipitation assay

293T cells were grown in 6-well plates and co-transfected with Flag-tagged TRIM16 and HA-tagged RIG-I plasmids using Lipofectamine 2000 (Invitrogen, USA). Cells were harvested 36 h post-transfection and lysed in Western/IP Cell Lysis Buffer (Biyuntian Biotechnology Co., Ltd., Shanghai, China). Cell lysates were incubated on ice for 40 minutes and then centrifuged at 12,000×g for 5 minutes at 4°C. The collected supernatants were prepared for subsequent analysis. Immunoprecipitation was performed with HA magnetic beads (Biyuntian, Shanghai, China). The immunoprecipitated complexes were washed thrice with lysis buffer, were eluted, and then analyzed by SDS-PAGE and Western blot using Flag and HA-specific antibodies.

### Ubiquitination assay

To characterize the direct E3 ligase activity of TRIM16 and its specific ubiquitin linkage in a cell-free system, recombinant human UbcH5b (HY-P79449, MedChemExpress) and RIG-I protein (TP317615, OriGene) were employed as the E2 conjugating enzyme and substrate, respectively. Full-length TRIM16, its B-box deletion mutant (ΔB-Box, residues 161–564), and ubiquitin mutants (K48-only and K63-only) were purified by Tsingke Biological Technology. The reaction mixture (50 µL) was assembled based on the Ubiquitylation Assay Kit (ab139467, Abcam) containing 100 nM E1 activating enzyme, 2.5 µM UbcH5b, 1 µM purified TRIM16 (WT or mutant), 0.5 µM RIG-I, and 50 µM of ubiquitin variants (WT, K48-only, or K63-only). Reactions were conducted in ubiquitination buffer supplemented with 20 U/mL inorganic pyrophosphatase (IPP; M0296S, New England Biolabs) and 1 mM DTT (D1070, Solarbio). The reaction was initiated by the addition of 5 mM Mg-ATP and incubated at 37°C for 2 hours. The process was terminated by boiling in reducing SDS-PAGE sample buffer. Ubiquitination levels were assessed by Western blotting using a specific anti-RIG-I antibody to visualize high-molecular-weight polyubiquitinated RIG-I smears [17].

To further validate the ubiquitin linkage specificity in a cellular context, HEK293T cells were seeded in 6-well plates and co-transfected with plasmids encoding HA-tagged RIG-I, Flag-tagged FGF8, and Myc-tagged ubiquitin variants (WT, K48R, or K63R) using Lipofectamine 2000. Twenty-four hours post-transfection, cells were treated with 10 µM MG132 (Selleck Chemicals, S2619) for 6 hours to block proteasomal degradation. Cells were then lysed in IP lysis buffer (Biyuntian Biotechnology) supplemented with protease inhibitors. The lysates were immunoprecipitated with Anti-HA-tag mAb-Magnetic Beads (MBL, M180-11) overnight at 4°C. The beads were washed five times with lysis buffer, and the immunoprecipitated complexes were eluted by boiling in SDS-PAGE sample buffer. The ubiquitination level of RIG-I was analyzed by Western blotting using anti-Myc antibody to detect ubiquitin chains and anti-HA antibody to detect total RIG-I.

### Protein stability and degradation pathway Analysis

A549 cells stably overexpressing FGF8 and the corresponding control cells were infected with H13N2 at an MOI of 1 for 6 hours. To evaluate RIG-I protein stability, a cycloheximide (CHX) chase assay was performed. Cycloheximide (Selleck Chemicals, S7418) was added at a final concentration of 50 μg/mL to inhibit new protein synthesis. Cells were collected at 0, 1, 2, 3, and 4 hours post-treatment for Western blot analysis. To investigate the degradation pathway, A549 cells were infected with influenza virus for 6 hours and subsequently treated for 6 hours with either the lysosomal inhibitor Chloroquine (50 µM, CQ; Selleck Chemicals, S6999) or the proteasome inhibitor MG132 (10 µM; Selleck Chemicals, S2619). Endogenous RIG-I protein levels were then examined by immunoblotting.

### Bioinformatic analysis of IDRs

The intrinsic disorder tendency of the FGF8 protein sequence was predicted using the Predictor of Natural Disordered Regions (PONDR) server (http://www.pondr.com). The analysis was performed using the VSL2 and VL3 predictors. Residues with a disorder score exceeding the threshold of 0.5 were considered to be located within intrinsically disordered regions (IDRs).

### TurboID-based proximity labeling assay

To characterize the interaction between FGF8 and TRIM16, HEK293T cells were transfected with pLV3-Flag-FGF8-TurboID using Lipofectamine 3000. Twenty-four hours post-transfection, cells were pre-treated with 10 µM MG132 for 4 h, followed by labeling with 50 µM D-Biotin (S3130, Selleck Chemicals) for 1.5 h at 37°C. Cells were lysed in Western and IP Cell Lysis Buffer, and biotinylated proteins were enriched using Streptavidin MagBeads (P0635S, Beyotime) overnight at 4°C. The beads were washed five times to remove nonspecific binding [18,19]. Bound proteins were eluted by boiling in SDS-PAGE sample buffer and analyzed by Western blotting using antibodies against TRIM16 and RIG-I.

### Determination of viral copy numbers

Viral copy numbers were determined using absolute quantitative real-time PCR (RT-qPCR). A standard curve was generated using ten-fold serial dilutions of a plasmid containing the NP gene. The viral copy number was calculated based on the cycle threshold (Ct) values using the linear regression equation derived from the standard curve: y = −0.3134x + 11.422, where y represents the logarithm of the copy number and ×represents the Ct value. The correlation coefficient (R^2^) was 0.9943.

### Isolation and culture of primary chicken embryo fibroblasts (CEFs)

Primary CEFs were isolated from embryonic day 10 (E10) specific pathogen-free (SPF) chicken embryos. Briefly, the embryos were decapitated, and the limbs and viscera were carefully removed. The remaining trunk tissues were washed with sterile phosphate-buffered saline (PBS) and minced into approximately 1 mm^3^ pieces using dissection scissors. The tissue fragments were digested with 0.25% Trypsin-EDTA at 37°C for 15 min with gentle agitation. The digestion was terminated by the addition of complete medium containing 10% FBS. The resulting cell suspension was filtered through a 70-µm cell strainer to remove tissue debris and centrifuged at 1,000 for 5 min. The cell pellet was resuspended in DMEM supplemented with 10% FBS and 1% penicillin-streptomycin. Cells were seeded into culture dishes and incubated at 37°C in a humidified atmosphere containing 5% CO2.

### Quantification and statistical analysis

Data were analyzed using GraphPad Prism 10.1.1 (GraphPad, San Diego, CA, USA). Comparisons between the two groups were performed using a two-tailed unpaired t-test, with the significance level set at *p* ≤ 0.05. Homogeneity of variance between groups was assessed using the F test. Results are presented as mean ± standard error of the mean (mean ± SEM), derived from at least three independent experiments. Statistical significance was defined as *p* ≤ 0.05.

## Results

### Gene expression analysis in A549 cells post-infection with H1N1 and H13N2 influenza virus

**Figure 1.**
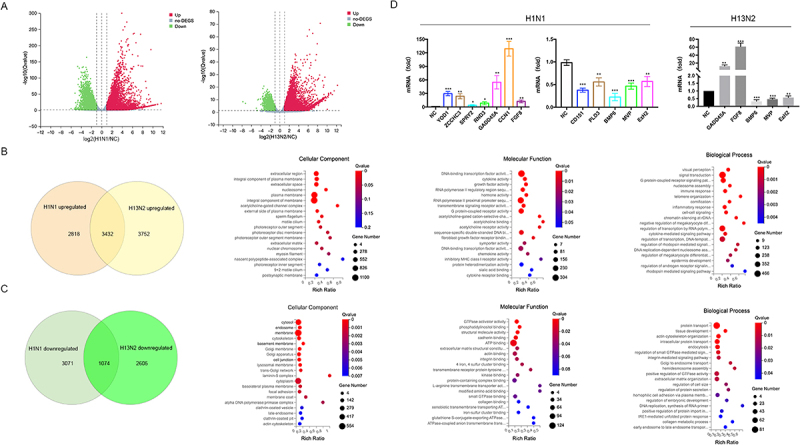
Analysis of A549 cell transcriptomes post-influenza a virus infection. (a) genes showing differential expression in A549 cells after a 24-hour infection with H1N1 or H13N2. (b) gene ontology (GO) enrichment analysis was conducted on genes commonly upregulated in A549 cells following infection with H1N1 and H13N2. (C) GO enrichment analysis of genes consistently downregulated in A549 cells following infection with H1N1 and H13N2. (d) transcriptomic data validation was conducted via RT-qPCR on selected differentially expressed genes in A549 cells infected with H1N1 or H13N2. Error bars indicate the mean±SEM from three independent experiments. Statistical significance was assessed using two-tailed unpaired Student’s t-tests, with thresholds set at *p < 0.05, **p < 0.01, and ***p < 0.001.

Transcriptomic analysis was conducted on A549 cells infected with H13N2 or H1N1 for 24 hours to examine the host’s transcriptional response. Differential gene expression analysis revealed that H1N1 infection resulted in 6,264 upregulated and 4,145 downregulated genes, while H13N2 infection induced 7,184 upregulated and 3,679 downregulated genes (Figure 1(A)). Comparative analysis of the two strains identified 3,432 commonly upregulated and 1,074 commonly downregulated genes (|log2| ≥1, Q-value ≤ 0.05, Figure 1(B, C)).

Gene Ontology (GO) enrichment analysis was performed for consistently up-regulated and down-regulated genes across three dimensions: Cellular Component (CC), Biological Process (BP), and Molecular Function (MF). In the CC category, the top three enriched terms for commonly upregulated genes were extracellular region, plasma membrane, and cell-cell junction, while for commonly downregulated genes, they were cytosol, endosome, and membrane. In the MF category, commonly upregulated genes were most enriched in terms such as specific DNA-binding transcription factor activity, cytokine activity, and growth factor activity. Conversely, for commonly downregulated genes, the top terms were GTPase activator activity, phosphatidylinositol binding, and structural constituent of cytoskeleton or organelles. In the BP category, commonly upregulated genes were enriched in visual perception, signal transduction, and G-protein coupled receptor signaling pathways, while commonly downregulated genes were enriched in protein transport, tissue development, and actin cytoskeleton organization (Figure 1(B, C)).

RT-qPCR was conducted on selected differentially expressed genes to validate the transcriptomic data. In H13N2-infected A549 cells, mRNA levels of GADD45A and FGF8 were significantly upregulated, while BMP6, MVP, and EZH2 were significantly downregulated. Similarly, in H1N1-infected cells, mRNA levels of CLDN4, GADD45A, YOD1, ZCCHC3, and FGF8 were significantly upregulated, while MVP and EZH2 were significantly downregulated (Figure 1(D)). These results confirmed the reliability of the transcriptomic data.

### The host factor FGF8 promotes influenza virus replication

**Figure 2.**
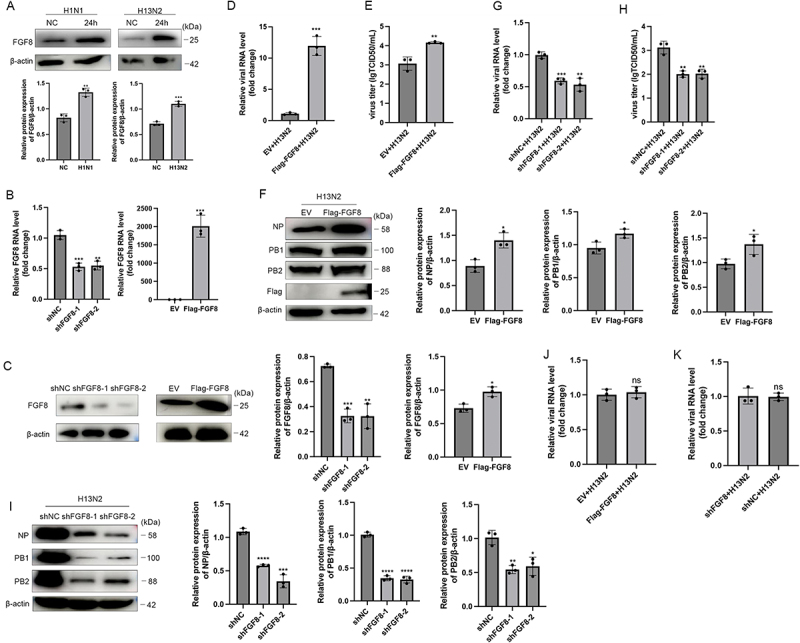
FGF8 promoted H13N2 influenza virus replication. (a) FGF8 expression in A549 cells was evaluated 24 hours after H1N1 or H13N2 infection (MOI = 0.5) using Western blot analysis, and band intensities were quantified by densitometry. (b, C) validation of FGF8 knockdown (shFGF8) and overexpression (Flag-FGF8) in A549 cell lines was conducted using RT-qPCR and Western blot. Relative protein levels were quantified by densitometric analysis. (D-F) Following 24 hours of H13N2 infection (MOI = 0.5) in A549 cells transiently transfected with FGF8 expression plasmids, viral RNA levels were measured by RT-qPCR (d), viral titers were determined using TCID50 assay (e), and Western blot analysis was performed to assess the expression of viral proteins NP, PB1, and PB2, with band intensities quantified by densitometry (f). (G-I) A549 cells with silenced FGF8 were infected with H13N2 for 24 hours, viral RNA levels were measured by RT-qPCR (G), viral titers were determined using TCID50 assay (H), and Western blot analysis was performed to assess the expression of viral proteins NP, PB1, and PB2, followed by densitometric analysis (i). (J and K) after 2 hours of H13N2 infection (MOI = 5) in A549 cells with FGF8 knockdown or overexpression, NP mRNA levels were detected using RT-qPCR. Error bars indicate the mean ± SEM from three independent experiments. Statistical analysis was performed using two-tailed unpaired Student’s t-tests, with significance thresholds defined as ns *p* > 0.05, *p < 0.05, **p < 0.01, and ***p < 0.001.

FGF8 expression was significantly upregulated in A549 cells post-infection with H13N2 or H1N1, as shown by both transcriptomic analysis and RT-qPCR (Figure 1(D)). Western blot analysis demonstrated a significant increase in FGF8 protein levels in A549 cells 24 hours after infection with either virus (Figure 2(A)). Further characterization revealed that this upregulation occurred in a dose- and time-dependent manner (Figure S1A).

To explore FGF8’s role in influenza virus replication, A549 cell lines with either FGF8 overexpression or knockdown were created and confirmed using RT-qPCR and Western blot (Figure 2(B, C)). After 24 hours of H13N2 infection, FGF8 overexpression markedly elevated NP mRNA levels, viral titers, and the expression of viral proteins NP, PB1, and PB2 (Figure 2(D-F)). In contrast, knockdown of FGF8 significantly reduced viral RNA levels (Figure 2(G)), viral titters (Figure 2(H)), and viral protein expression (Figure 2(I)). The results indicate that FGF8 potentially enhances viral replication and infection.

To determine whether the proviral role of FGF8 is broad-spectrum, we extended our investigation to other influenza A virus subtypes, including H1N1 (swine origin), H9N2 (avian origin), and PR8 strain. Consistent with our initial findings, infection with H9N2 or PR8 significantly upregulated FGF8 expression (Figure S1B, C). Functionally, in H1N1-infected cells, FGF8 overexpression significantly increased viral NP mRNA levels, titers, and NP protein expression, whereas FGF8 knockdown markedly suppressed these parameters (Figure S1D). Consistently, Western blot analysis of H9N2- or PR8-infected cells revealed that FGF8 overexpression upregulated, while knockdown downregulated, the levels of multiple viral proteins, including NP, PB1, and PB2 (Figure S1E-H). Additionally, we evaluated FGF8 expression in CEFs to assess host specificity. While H1N1, H9N2, and H13N2 replicated efficiently in CEFs, FGF8 mRNA levels were downregulated (H9N2 and H13N2) or remained unchanged (H1N1) following infection, contrasting with the upregulation observed in human A549 cells (Figure S1J, K).

To evaluate FGF8’s effect on viral entry, A549 cells with either overexpressed or knocked down FGF8 were exposed to influenza virus on ice for 30 minutes, followed by a 2-hour incubation at 37°C. Cells were washed with pH 1.3 PBS to eliminate uninternalized virus particles. RT-qPCR analysis showed that FGF8 overexpression or knockdown did not affect viral endocytosis (Figure 2(J, K) and Figure S1I). These results suggest that FGF8 enhances influenza A virus replication without influencing viral entry.

### FGF8 inhibits type I interferon signaling triggered by influenza virus infection

In A549 cells infected with H13N2, the transcription levels of RIG-I, MDA5, IFN-β, MX1, and IFIT1 were upregulated, while MAVS expression was downregulated (Figure S2A-F). Furthermore, infection with other influenza A virus subtypes, including H1N1, H9N2, and PR8, also induced significant upregulation of RIG-I at both mRNA and protein levels (Figure S2G).

**Figure 3.**
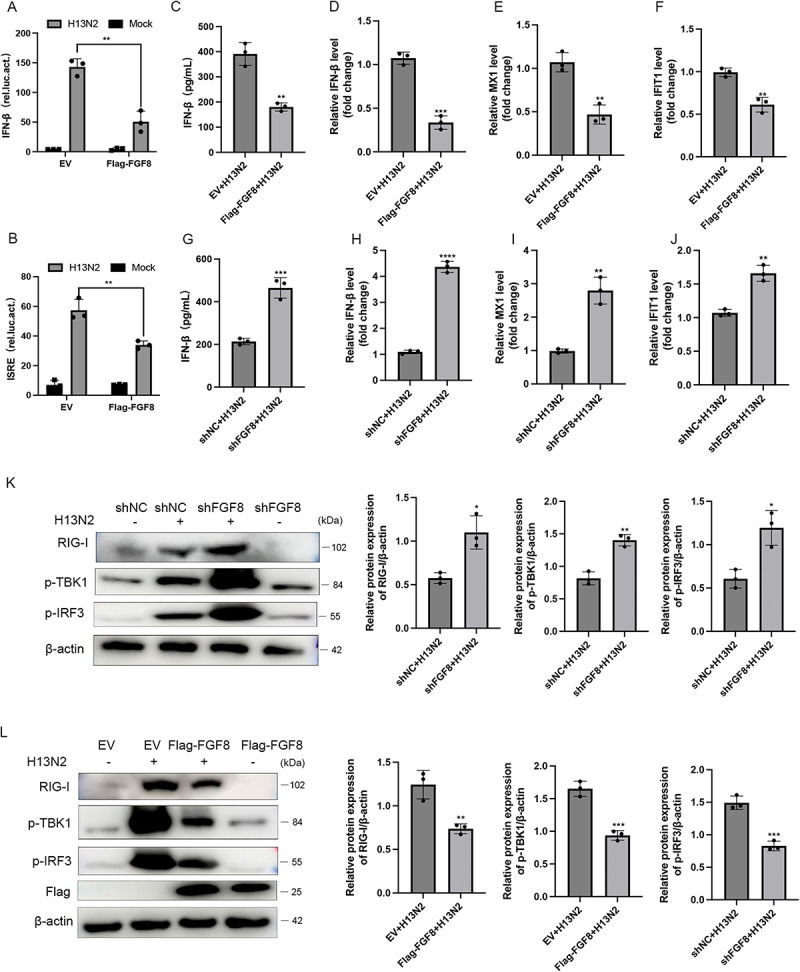
FGF8 negatively regulated IFN-β induced by H13N2 infection. (a, B) luciferase reporter assays were used to assess the impact of FGF8 overexpression on IFN-β and ISRE promoter activity in A549 cells infected with H13N2 at an MOI of 1. (C-F) FGF8-overexpressing A549 cells were infected with H13N2 at an MOI of 1. At 12 hours post-infection (hpi), IFN-β levels in the cell supernatant were measured using ELISA (C), and IFN-β mRNA levels were evaluated by RT-qPCR (d). At 24 hpi, the mRNA levels of interferon-stimulated genes MX1 (e) and IFIT1 (f) were assessed by RT-qPCR. (G-J) stable FGF8-knockdown A549 cells were infected with H13N2 at an MOI of 1. At 12 hpi, IFN-β levels in the cell supernatant were quantified by ELISA (G), and IFN-β mRNA levels were evaluated using RT-qPCR (H). At 24 hpi, the mRNA levels of MX1 (i) and IFIT1 (J) were assessed by RT-qPCR. (K and L) Western blot analysis evaluated RIG-I, p-TBK1, and p-IRF3 expression in A549 cells with FGF8 overexpression (L) or knockdown (K) at 12 hours after H13N2 infection (MOI = 1). Band intensities were quantified by densitometric analysis. Statistical analysis was performed using two-tailed unpaired Student’s t-tests, with significance levels of *p < 0.05, **p < 0.01, and ***p < 0.001.

In H13N2-infected cells, FGF8 overexpression notably inhibited IFN-β and ISRE promoter activity (Figure 3(A, B)). FGF8 overexpression also reduced H13N2-induced IFN-β expression (Figure 3(C,D)) and significantly downregulated the mRNA levels of MX1 and IFIT1 (Figure 3(E,F)). Conversely, FGF8 knockdown enhanced H13N2-induced IFN-β expression and upregulated MX1 and IFIT1 mRNA levels (Figure 3(G-J)).

Western blot analysis revealed that FGF8 knockdown elevated RIG-I expression and increased TBK1 and IRF3 phosphorylation, whereas FGF8 overexpression produced contrary results (Figure 3(K,L)). Comparable findings were noted in cells infected with H1N1 (Figure S3A-I). These findings suggest that FGF8 suppresses type I interferon signaling by modulating RIG-I expression.

### FGF8 promotes RIG-I degradation via the ubiquitin-proteasome pathway

**Figure 4.**
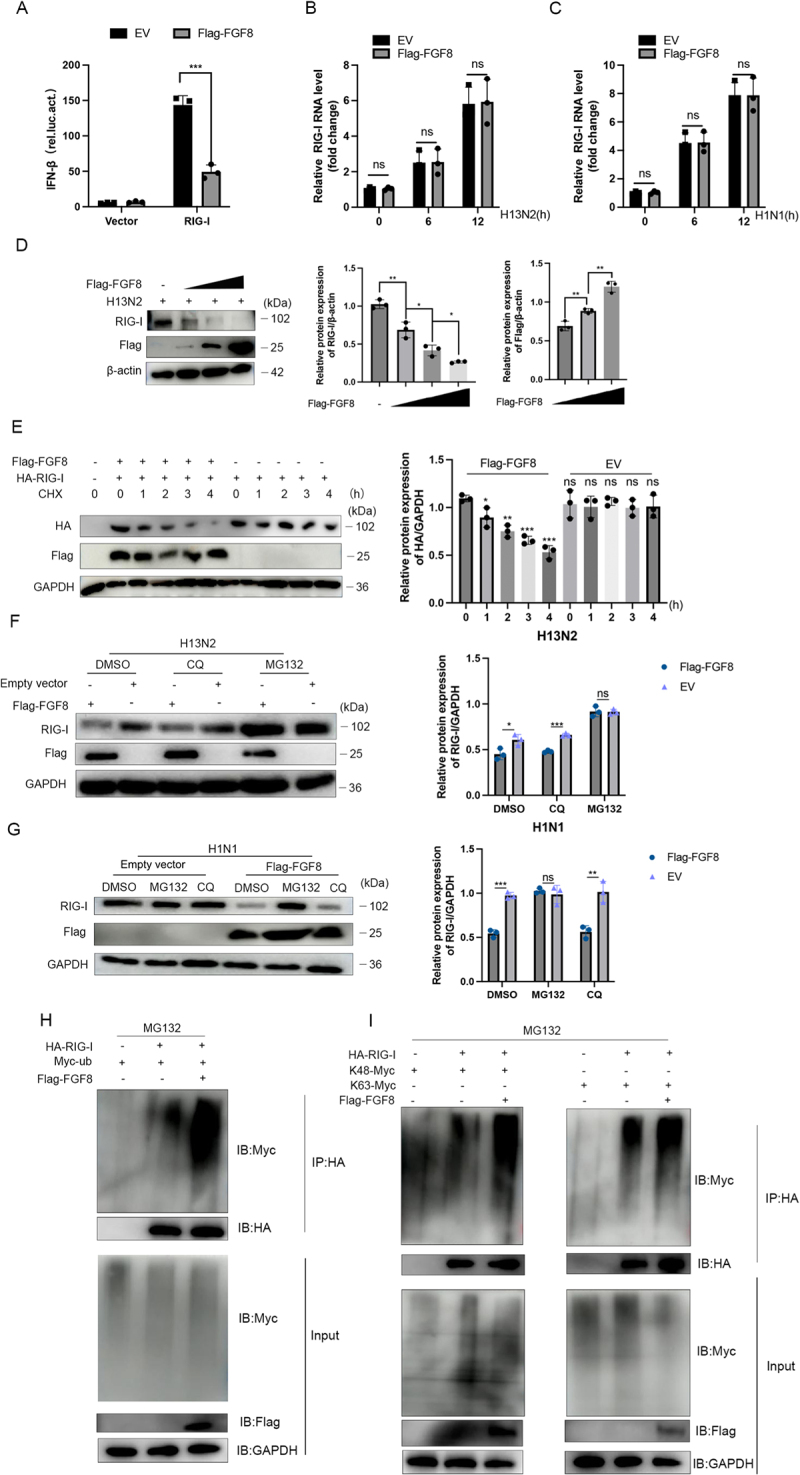
FGF8 drives ubiquitin – proteasomal degradation of RIG-I. (a) FGF8 inhibits RIG-I-mediated signaling. A luciferase reporter assay was performed to evaluate the effect of FGF8 overexpression on IFN-β promoter activation induced by RIG-I. (B and C) FGF8 does not affect RIG-I transcription. RIG-I mRNA levels were quantified by RT-qPCR in FGF8-overexpressing A549 cells at 0, 6, and 12 hours post-infection with H13N2 (b) or H1N1 (C) at an MOI of 1. (d) dose-dependent reduction of RIG-I protein. A549 cells were transfected with increasing amounts of Flag-FGF8 plasmid for 24 hours, followed by infection with H13N2 (MOI = 1) for 12 hours. RIG-I protein levels were analyzed by Western blot, and band intensities were quantified by densitometry. (e) FGF8 reduces RIG-I stability. FGF8-overexpressing A549 cells were infected with H13N2 (MOI = 1) and treated with cycloheximide (CHX, 50 µg/mL) for the indicated time periods. Protein levels were analyzed by Western blot, and the relative abundance of HA-RIG-I was quantified to assess protein degradation rates. (F and G) proteasome inhibition restores RIG-I levels. A549 cells infected with H13N2 (f) or H1N1 (G) at an MOI of 1 were treated with DMSO, chloroquine (CQ, 50 µM), or MG132 (10 µM) for 6 hours. RIG-I expression was analyzed by Western blot, with relative protein levels quantified by densitometry. (H and I) FGF8 promotes K48-linked ubiquitination of RIG-I. HEK-293T cells were co-transfected with the indicated plasmids and treated with MG132 for 6 hours. (H) Total ubiquitination of RIG-I was assessed by immunoprecipitation with anti-HA antibody followed by immunoblotting (ib) with anti-Myc. (i) K48- or K63-linked ubiquitination was analyzed using specific ubiquitin mutants. Error bars indicate the mean ± SEM from three independent experiments. Statistical analysis was performed using two-tailed unpaired Student’s t-tests. ns (not significant), *p < 0.05, **p < 0.01, and ***p < 0.001.

Luciferase reporter assays revealed that FGF8 overexpression significantly inhibited RIG-I-induced activation of the IFN-β promoter (Figure 4(A)). Notably, FGF8 overexpression did not affect RIG-I mRNA levels in H13N2- or H1N1-infected cells (Figure 4(B, C)), suggesting that FGF8 regulated RIG-I expression at the post-translational level. As FGF8 expression increased, RIG-I protein levels decreased in a dose-dependent manner (Figure 4(D)).

Cycloheximide (CHX) treatment revealed that FGF8 accelerated RIG-I protein degradation (Figure 4(E)). FGF8-induced RIG-I degradation was significantly inhibited by MG132 but not by CQ, indicating that FGF8 promotes RIG-I degradation through the proteasome pathway (Figure 4(F, G)).

Ubiquitination assays demonstrated that FGF8 overexpression notably enhanced RIG-I ubiquitination, particularly K48-linked ubiquitination, which is linked to proteasome-mediated degradation (Figure 4(H, I)).

### Lysine 258 of RIG-I is ubiquitinated during FGF8-mediated degradation

**Figure 5.**
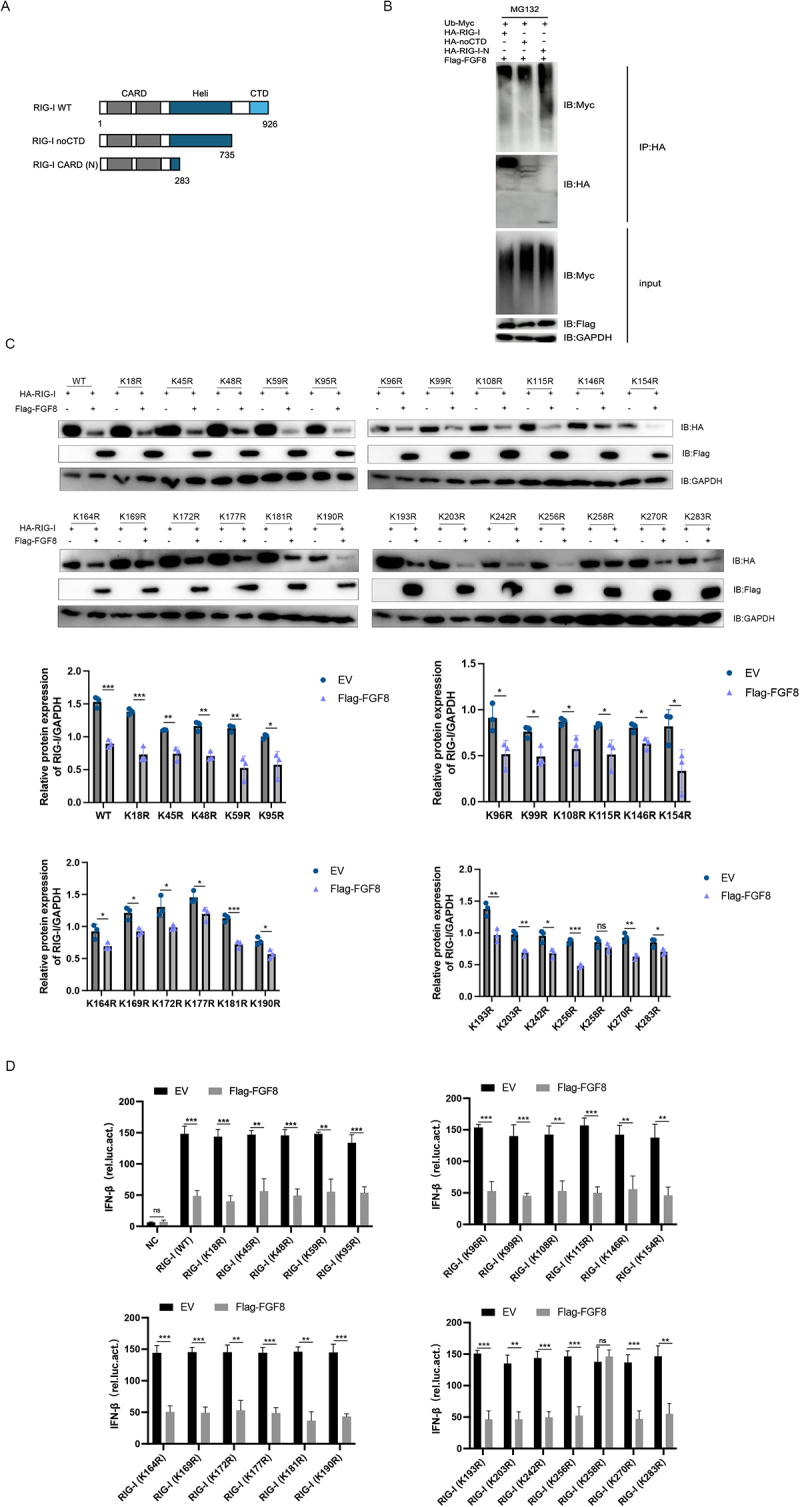
Identification of the ubiquitination site on RIG-I targeted by FGF8. (a) diagram illustrating the truncated constructs of RIG-I. (b) HEK-293T cells were co-transfected with specified plasmids and exposed to MG132 for 6 hours. Western blot analysis was conducted to assess the ubiquitination of various RIG-I truncation constructs. (C) Western blot analysis identified the ubiquitination site on RIG-I targeted by FGF8, and band intensities were quantified by densitometry to assess the degradation of each mutant. (d) a dual-luciferase assay was conducted in HEK293T cells co-transfected with specified RIG-I mutants and FGF8 to evaluate the impact of FGF8 on IFN-β promoter activity. Error bars indicate the mean ± SEM from three independent experiments. Two-tailed unpaired Student’s t-tests were used. ns (not significant), *p < 0.05, **p < 0.01, and ***p < 0.001.

To determine the specific domain of RIG-I targeted by FGF8, constructs of full-length RIG-I (RIG-I WT), a C-terminal domain-lacking mutant (RIG-I noCTD), and a mutant with only the CARD domain (RIG-I CARD (N)) were created (Figure 5(A)). Ubiquitination assays showed that all constructs were ubiquitinated in the presence of FGF8, indicating that the CARD domain is critical for FGF8-mediated ubiquitination (Figure 5(B)).

Mutational analysis of lysine residues in RIG-I identified lysine 258 as the key site for FGF8-induced ubiquitination. The mutation of lysine 258 inhibited RIG-I degradation (Figure 5(C)) and counteracted the suppressive impact of FGF8 on RIG-I-mediated activation of IFN-β transcription (Figure 5(D)).

### FGF8 recruits TRIM16 to mediate RIG-I degradation and promote influenza virus replication

**Figure 6.**
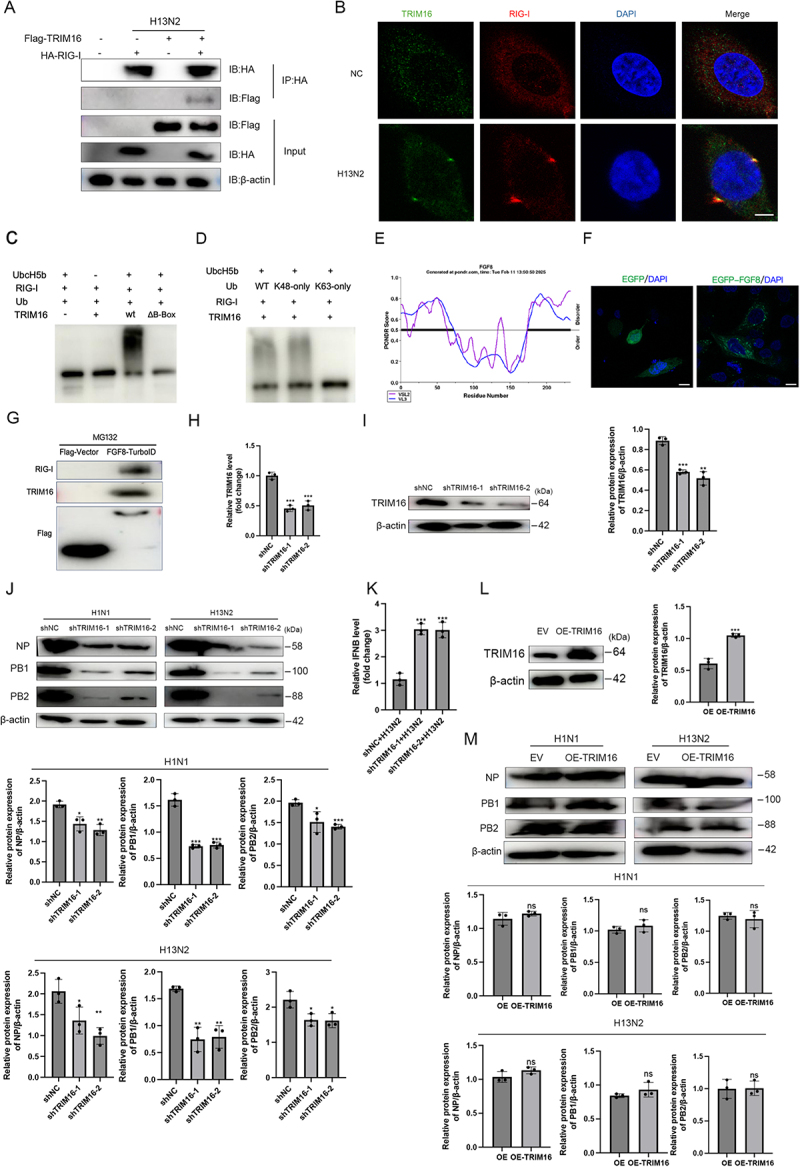
TRIM16 mediated RIG-I degradation and promoted influenza virus replication. (a) Co-immunoprecipitation analysis was performed in cells transfected with Flag-TRIM16 and HA-RIG-I, with or without H13N2 infection (MOI = 1), to verify the interaction. (b) immunofluorescence microscopy showing the localization of TRIM16 (green) and RIG-I (red) in cells infected with H13N2 or mock-infected (NC). Nuclei were stained with DAPI (blue). Note that TRIM16 and RIG-I show diffuse distribution in the NC group but form co-localized puncta (yellow) upon H13N2 infection. Scale bar: 5 μm. (C) *in vitro* ubiquitination assay to verify the direct E3 ligase activity of TRIM16 using wt and ΔB-Box mutant proteins. (d) *in vitro* ubiquitination assay to determine the linkage specificity of TRIM16-mediated RIG-I ubiquitination using K48-only and K63-only ubiquitin mutants. (e) bioinformatic analysis using PONDR revealed the presence of intrinsically disordered regions (IDRs) in the FGF8 protein sequence. (f) fluorescence microscopy of A549 cells transfected with EGFP-FGF8 (green). Nuclei were stained with DAPI. Scale bar represents 10 μm. (G) TurboID-based proximity labeling assay was performed in cells expressing FGF8-TurboID. Biotinylated proteins were captured using streptavidin beads, and the pulled-down proteins were analyzed by Western blot to detect the presence of RIG-I and TRIM16. (H and I) validation of TRIM16 knockdown. RT-qPCR (H) and Western blot (i) confirmed the silencing efficiency in A549 cells. (J) control and TRIM16-silenced A549 cells were infected with H1N1 or H13N2 (MOI = 0.5) for 24 hours. Viral protein levels (NP, PB1, PB2) were analyzed by Western blot, and band intensities were quantified by densitometry. (K) RT-qPCR analysis of IFN-β mRNA levels in TRIM16-silenced A549 cells 12 hours post-infection with H13N2 (MOI = 1). (L) Western blot confirmation of TRIM16 overexpression (OE-TRIM16). (M) A549 cells overexpressing TRIM16 were infected with H1N1 or H13N2 (MOI = 0.5) for 24 hours. Viral protein expression was analyzed by Western blot and quantified by densitometry. Error bars indicate the mean ± SEM from three independent experiments. Statistical analysis was performed using two-tailed unpaired Student’s t-tests. ns (not significant), *p < 0.05, **p < 0.01, and ***p < 0.001.

Immunoprecipitation coupled with mass spectrometry revealed that TRIM16 functions as an E3 ubiquitin ligase interacting with RIG-I in the context of influenza virus infection. Co-immunoprecipitation experiments confirmed the interaction between TRIM16 and RIG-I (Figure 6(A)). We next examined the subcellular localization of TRIM16 and RIG-I using confocal microscopy. In uninfected cells (NC), TRIM16 and RIG-I displayed a dispersed distribution pattern throughout the cytoplasm. In contrast, H13N2 infection triggered a marked redistribution of both proteins, leading to their co-localization in speckle-like structures (Figure 6(B)).

To definitively determine whether TRIM16 acts as a direct E3 ubiquitin ligase for RIG-I, we performed an *in vitro* ubiquitination assay using a reconstituted cell-free system with purified recombinant proteins. As shown in Figure 6(C), in the presence of E1, E2 (UbcH5b), and ATP, wild-type (WT) TRIM16 induced robust polyubiquitination of RIG-I, whereas the B-box deletion mutant (ΔB-Box) failed to do so. Furthermore, consistent with our cellular findings, TRIM16 directly catalyzed RIG-I ubiquitination in the presence of WT and K48-only ubiquitin but failed to utilize K63-only ubiquitin (Figure 6(D)). These biochemical data provide direct evidence that TRIM16 functions as an E3 ligase that specifically catalyzes the K48-linked polyubiquitination of RIG-I.

To further elucidate how FGF8 coordinates this process, we attempted to detect the interaction between FGF8 and TRIM16. However, standard Co-IP assays failed to reveal a stable complex between these two proteins (data not shown). Suspecting that the interaction might be weak or transient, we analyzed the structural characteristics of FGF8. Bioinformatic analysis using PONDR revealed that FGF8 contains distinct intrinsically disordered regions (IDRs) (Figure 6(E)), and fluorescence microscopy showed that FGF8 forms cytoplasmic puncta (Figure 6(F)). These features suggest that FGF8 may engage in dynamic spatial clustering rather than forming rigid static complexes. To capture such proximal interactions in living cells, we employed a TurboID-based proximity labeling assay. As shown in Figure 6(G), streptavidin pulldown of biotinylated proteins from FGF8-TurboID expressing cells resulted in the significant enrichment of both TRIM16 and RIG-I compared to the control. These data demonstrate that despite the lack of stable binding in Co-IP, FGF8 resides in close spatial proximity to the TRIM16-RIG-I complex, likely facilitating the ubiquitination process by recruiting them into these cytoplasmic structures.

Knockdown of TRIM16 significantly inhibited influenza virus replication (Figure 6(H-J)) and increased virus-induced IFN-β production (Figure 6(K)). However, overexpression of TRIM16 did not significantly affect virus replication (Figure 6(L, M)). These results suggested that TRIM16 mediated RIG-I degradation to facilitate influenza virus replication.

### FGF8 is upregulated by VSV infection and promotes viral replication

**Figure 7.**
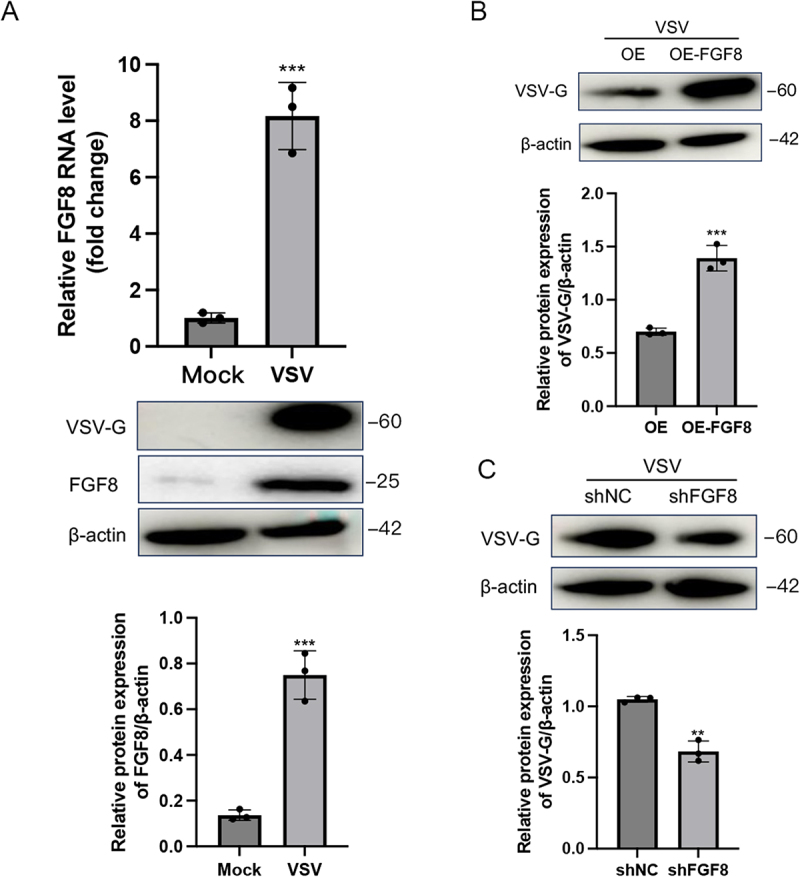
FGF8 is upregulated by VSV infection and promotes viral replication. (a) upregulation of FGF8 by VSV infection. A549 cells were infected with VSV at an MOI of 0.5 for 24 h. FGF8 mRNA levels were determined by RT-qPCR, and protein expression was analyzed by Western blot, with band intensities quantified by densitometry. (B and C) effect of FGF8 on VSV replication. A549 cells with FGF8 overexpression (b) or knockdown (C) were infected with VSV at an MOI of 0.5 for 24 h. The expression levels of the viral protein VSV-G were determined by Western blot, and relative protein levels were quantified by densitometric analysis. Data are presented as mean ± SEM from three independent experiments. Statistical analysis was performed using two-tailed unpaired Student’s t-tests. *p < 0.05, **p < 0.01, and ***p < 0.001.

We further explored whether this mechanism applies to other RNA viruses recognized by RIG-I, such as VSV. Similarly, both RT-qPCR and Western blot analyses revealed that VSV infection significantly upregulated FGF8 expression (Figure 7(A)). Functionally, FGF8 knockdown decreased, whereas FGF8 overexpression increased, the protein levels of VSV-G (Figure 7(B, C)). These data suggest that FGF8 functions as a broad-spectrum proviral host factor that facilitates the replication of diverse RNA viruses.

## Discussion

FGF8 is a secreted signaling protein known for its essential roles in embryonic development and tumorigenesis [7,20,21]. However, its function in viral infection remains largely unexplored. This study is the first to demonstrate that FGF8 acts as a potent proviral host factor that markedly boosts influenza A virus replication, offering a novel perspective on its non-canonical role in innate immunity.

The innate immune system relies on sensors like RIG-I to detect viral RNA and trigger type I interferon (IFN) signaling [22,23]. However, influenza viruses can also evade or suppress innate immune responses through various mechanisms. The NS1 protein of the influenza virus interacts with 14–3-3 proteins, interfering with the RIG-I and mitochondrial antiviral-signaling protein (MAVS) interaction, which leads to the suppression of type I interferon production [24]. The virus can also affect host cell functions via other mechanisms, like the PB1-F2 protein of the influenza virus, which induces mitochondrial membrane potential depolarization, resulting in cell apoptosis and facilitating viral release and spread [25]. The influenza virus NS1 binds directly to RIG-I, obstructing its interaction with viral RNA and subsequently inhibiting RIG-I activation, which diminishes the host’s antiviral immune response [26]. The Ebola virus VP35 protein disrupts RIG-I function by competing for RNA binding, aiding the virus in evading immune detection [27]. Host proteins regulate RIG-I activity, with TRIM25 enhancing it via K63-linked ubiquitination to boost antiviral signaling, whereas USP21 diminishes RIG-I activity by removing these ubiquitin chains, thus weakening the antiviral immune response [28]. Our study reveals that FGF8 functions as a hijacked host regulator in this context. We demonstrated that FGF8 overexpression significantly inhibits IAV-induced IFN-β and ISG expression, thereby promoting viral replication by downregulating RIG-I protein levels.

Mechanistically, we identified that FGF8 promotes RIG-I degradation via the TRIM16-mediated ubiquitin-proteasome pathway. TRIM16 is a multifunctional E3 ubiquitin ligase crucial for cell cycle regulation, inflammation control, and antiviral defense. It modifies target proteins through ubiquitination, marking them for degradation via the proteasome pathway [29,30]. TRIM16 regulates NLR family pyrin domain containing 3 (NLRP3) inflammasome assembly during inflammation to promote IL-1β release [31], and modulates antiviral functions by ubiquitinating RIG-I or other immune proteins [32]. In this study, we discovered that TRIM16 interacts with RIG-I, and silencing TRIM16 significantly inhibited influenza virus replication while increasing virus-induced IFN-β production. Furthermore, our *in vitro* ubiquitination assays using purified proteins provided direct biochemical evidence that TRIM16 functions as an E3 ligase to catalyze K48-linked polyubiquitination of RIG-I. These findings enhance our comprehension of TRIM16’s role in viral infections.

To further elucidate the mechanism by which FGF8 regulates TRIM16, we analyzed the structural characteristics of the FGF8 protein. Emerging evidence suggests that proteins containing IDRs often drive liquid-liquid phase separation (LLPS) to form biomolecular condensates, which serve as “reaction crucibles” to concentrate enzymes and substrates for efficient ubiquitination [33–37]. Interestingly, we identified distinct IDRs within FGF8 and observed its formation of cytoplasmic puncta. Although traditional Co-IP assays did not detect a stable interaction among FGF8, TRIM16, and RIG-I, our TurboID proximity labeling results confirmed that these proteins reside in close proximity within living cells [18]. Based on these findings, we speculate that FGF8 may facilitate the ubiquitination of RIG-I by recruiting TRIM16 into condensates. It should be noted that while these features are characteristic of LLPS, the precise biophysical properties of these condensates remain to be fully characterized by methods such as Fluorescence Recovery After Photobleaching (FRAP), which will be an important direction for our future research.

Given the absence of TRIM16 in avian hosts and the lack of FGF8 upregulation in infected avian cells, this degradation mechanism appears to be unique to the mammalian immune environment. This suggests that influenza viruses exploit the FGF8-mediated recruitment of TRIM16 to counteract the potent RIG-I response upon crossing into mammalian hosts. Importantly, we observed that FGF8 expression was consistently upregulated by diverse IAV subtypes. Crucially, FGF8 facilitated the replication of all these viral strains. This study further reveals that this FGF8-mediated degradation of RIG-I is not limited to specific influenza strains but may represent a broad-spectrum immune evasion strategy among influenza A viruses. Beyond influenza A viruses, we also observed that VSV infection led to an increase in FGF8 expression. Moreover, overexpression or knockdown of FGF8 resulted in an increase or decrease, respectively, in the protein levels of VSV-G. However, it is important to note that the detailed molecular mechanisms underlying the interplay between FGF8 and VSV were not further explored in the current study. Therefore, the precise basis of this interaction warrants further in-depth investigation in future studies.

In conclusion, this study demonstrates that FGF8 enhances viral replication by promoting RIG-I degradation through TRIM16-mediated ubiquitination, which in turn suppresses the type I interferon signaling pathway. This discovery not only deepens our understanding of the immune evasion mechanisms of influenza A viruses but also highlights FGF8 and TRIM16 as promising targets for the development of host-directed antiviral therapeutics against influenza infections.

## Supplementary Material

Supplementary materials revised.docx

## Data Availability

The data that support the findings of this study are openly available in Figshare at https://figshare.com/articles/dataset/Raw_data/30042133, reference number [38]. The RNA-seq data generated from A549 cells infected with H13N2 are openly available in GEO at https://www.ncbi.nlm.nih.gov/geo/query/acc.cgi?acc=GSE309539, reference number [39]. The RNA-seq data generated from A549 cells infected with H1N1 are openly available in NCBI BioProject at https://www.ncbi.nlm.nih.gov/sra/PRJNA1335521, reference number [40].
