# Supplementary material for: FGF8-mediated TRIM16 regulation promotes K48-linked ubiquitination and degradation of RIG-I to facilitate Influenza a virus immune evasion
Source: Virulence. 2026 May 22;17(1):2677346. doi: 10.1080/21505594.2026.2677346 (PMC13203027; doi:10.1080/21505594.2026.2677346)
Supplement: Supplementary materials revised.docx [file KVIR_A_2677346_SM4503.docx]

**Supplementary Materials**

**
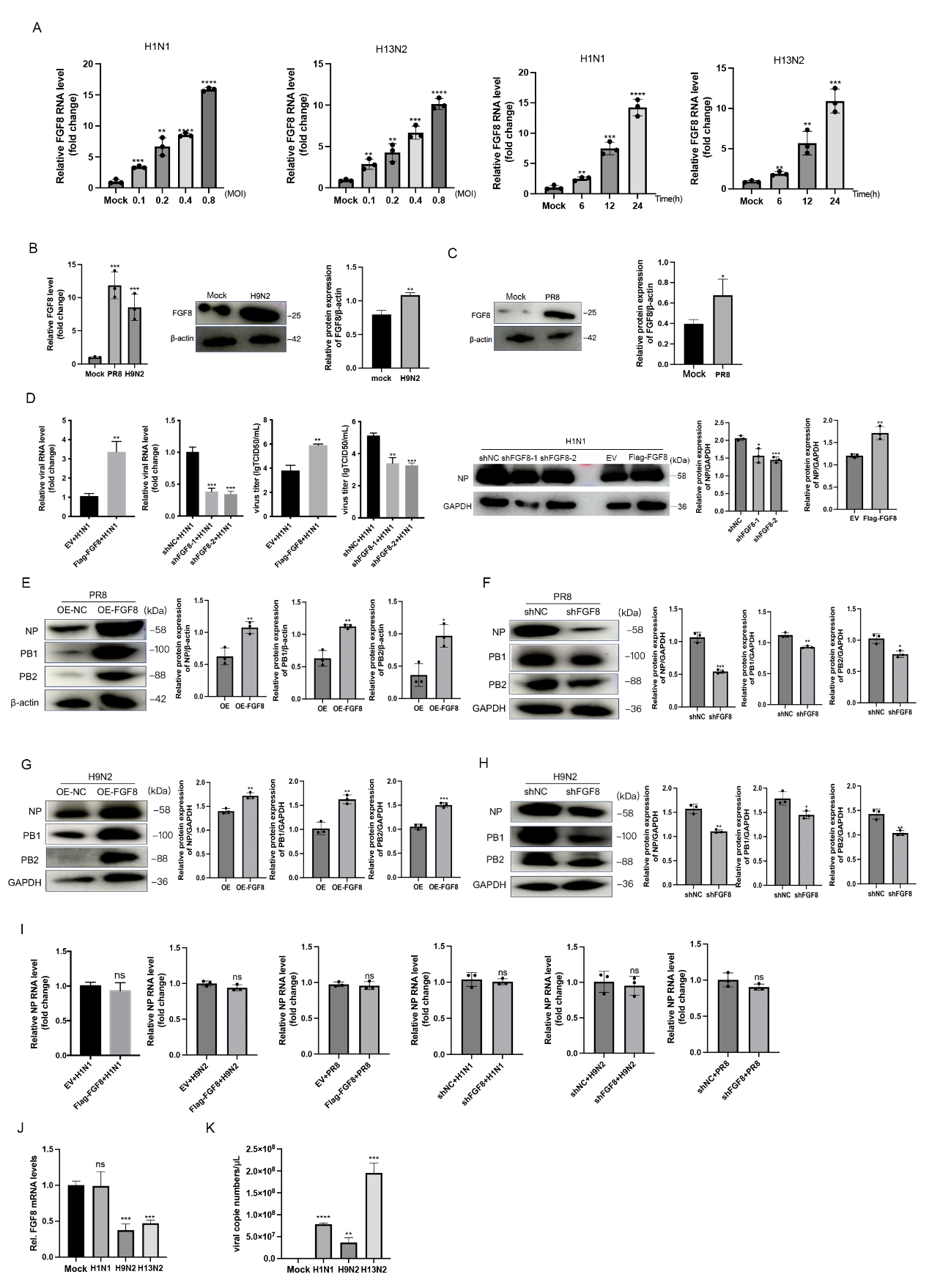
**

**Figure S1.** Broad-spectrum proviral role of FGF8 in influenza A virus replication. (A) Dose- and time-dependent upregulation of FGF8 mRNA. A549 cells were infected with influenza virus at indicated MOIs or at an MOI of 0.5 for different time points, and FGF8 transcript levels were determined by RT-qPCR. (B and C) Upregulation of FGF8 by H9N2 and PR8 subtypes. A549 cells were infected with H9N2 (B) or PR8 (C) strains at an MOI of 0.5. FGF8 expression levels were assessed by RT-qPCR and Western blot. Relative protein levels were quantified by densitometry. (D) FGF8 promotes H1N1 replication. A549 cells transiently transfected with FGF8 expression plasmids or stable FGF8-knockdown cell lines were infected with H1N1 (swine origin) at an MOI of 0.5. Viral NP mRNA levels, viral titers, and viral protein expression (analyzed by Western blot and densitometry) were evaluated at 24 hpi. (E to H) FGF8 regulates the replication of PR8 and H9N2 strains. A549 cells transiently transfected with FGF8 expression plasmids or stable FGF8-knockdown cell lines were infected with PR8 (E and F) or H9N2 (G and H) at an MOI of 0.5. The expression levels of viral proteins (NP, PB1, and PB2) were determined by Western blot, and band intensities were quantified by densitometric analysis. (I) FGF8 does not affect viral entry. A549 cells with manipulated FGF8 expression (transient overexpression or stable knockdown) were incubated with H1N1, H9N2, or PR8 at an MOI of 5 on ice for 30 min followed by 2 h at 37°C. Internalized viral RNA (NP) was quantified by RT-qPCR. (J and K) FGF8 expression and viral replication in avian cells. CEF cells were infected with H1N1, H9N2, or H13N2 at an MOI of 0.5 for 24 h. FGF8 mRNA levels (J) and viral copy numbers (K) were determined by RT-qPCR. Data are presented as mean ± SEM from three independent experiments. Statistical significance was determined using two-tailed unpaired Student’s t-tests. ns, not significant; *p < 0.05, **p < 0.01, ***p < 0.001, and ****p < 0.0001.


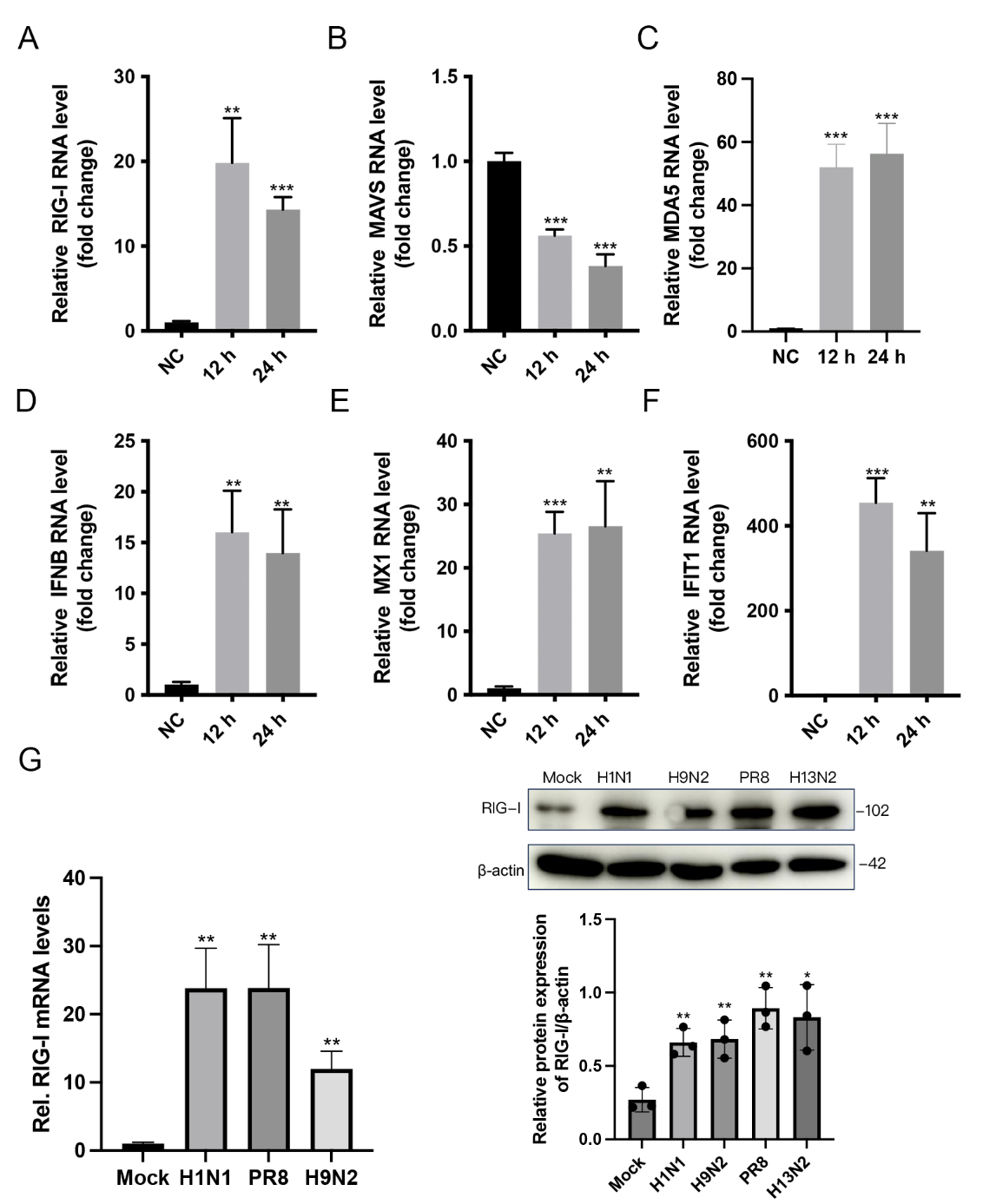


**Figure S2.** Expression of key proteins in the IFN-β signaling pathway after H13N2 infection in A549 cells. (A-D) RIG-I, MAVS, MDA5, and IFN-β mRNA expression at 12 h post H13N2 infection were detected by RT-qPCR. (E and F) RT-qPCR was conducted to assess the mRNA expression levels of MX1 and IFIT1 in A549 cells 24 hours post-H13N2 infection. (G) Upregulation of RIG-I by multiple influenza subtypes. A549 cells were infected with H1N1, PR8, H9N2, or H13N2 strains at an MOI of 1. RIG-I mRNA levels were measured by RT-qPCR, and protein expression was analyzed by Western blot, with band intensities quantified by densitometry. Error bars indicate the mean ± SEM from three independent experiments. Statistical analysis was performed using two-tailed unpaired Student's t-tests. *p < 0.05, **p < 0.01, and ***p < 0.001.

**
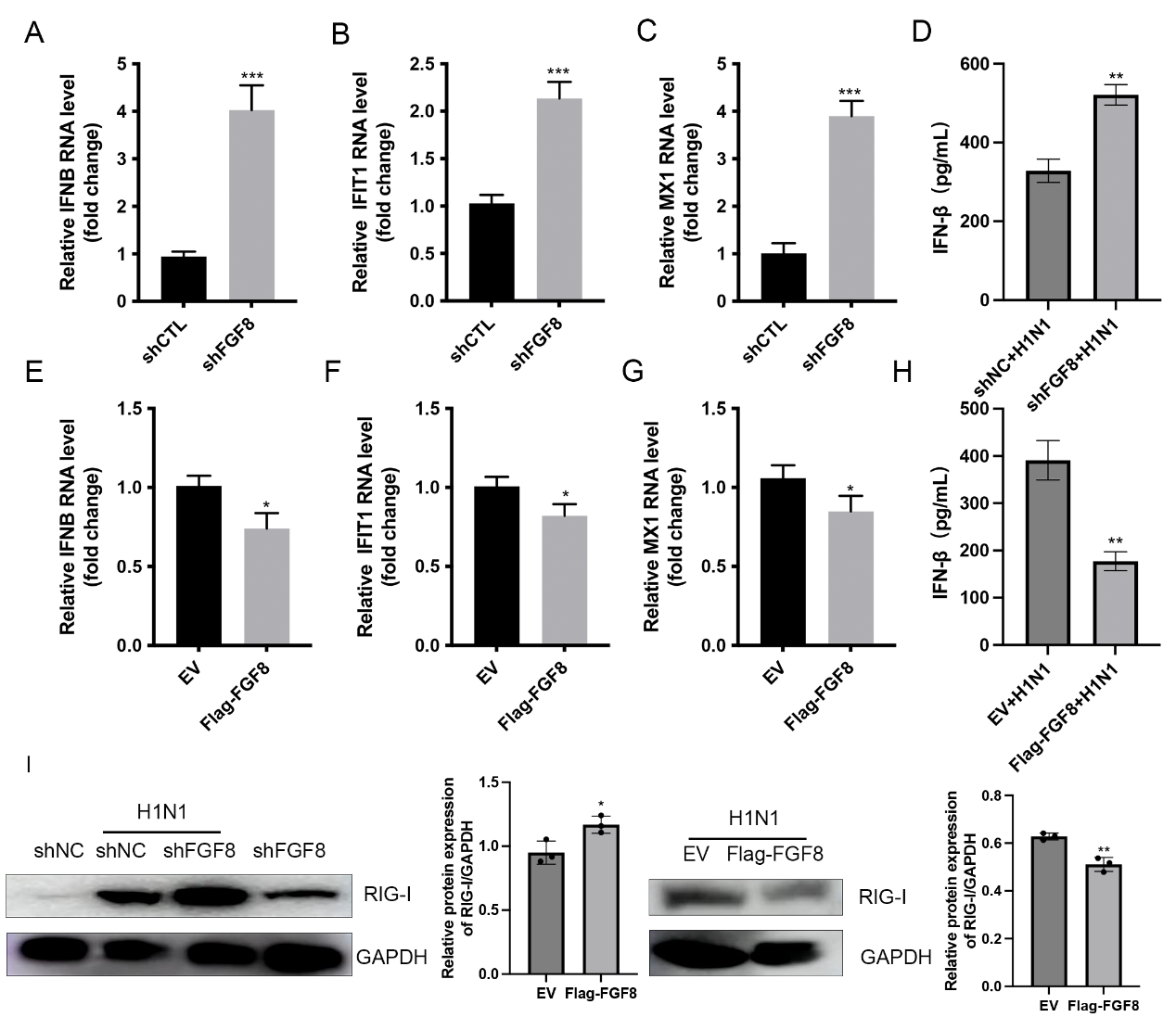
**

**Figure S3.** FGF8 negatively regulated type I interferon signaling induced by H1N1 influenza virus infection. A549 cells with FGF8 knockdown were infected with H1N1 (MOI=1) for 12 hours (A and D) or 24 hours (B and C). IFN-β levels in the cell supernatant were quantified by ELISA (D), and mRNA levels of IFN-β (A), MX1 (B), and IFIT1 (C) were evaluated using RT-qPCR. (E-H) FGF8 overexpression inhibits H1N1-induced signaling. A549 cells overexpressing FGF8 were infected with H1N1 (MOI=1) for 12 hours (E and H) or 24 hours (F and G). IFN-β levels in the cell supernatant were measured via ELISA (H), and RT-qPCR was used to evaluate mRNA levels of IFN-β (E), IFIT1 (F), and MX1 (G). (I) Western blot analysis was conducted to assess RIG-I expression in A549 cells with FGF8 overexpression or knockdown, 12 hours post-H1N1 infection (MOI=1). Relative protein levels were quantified by densitometry. Error bars indicate the mean ± SEM from three independent experiments. Two-tailed unpaired Student's t-tests were conducted for statistical analysis, with significance levels set at *p < 0.05, **p < 0.01, and ***p < 0.001.

**Table S1.** Primer sequences used in RT-qPCR.

| **Primer Name** | **Sequence (5’-3’)** |
| --- | --- |
| FGF8-F | GTGGAGACGGACACCTTTGG |
| FGF8-R | TGCTCTTGGCGATCAGCTT |
| GAPDH-F | ACCCACTCCTCCACCTTTGA |
| GAPDH-R | TGTTGCTGTAGCCAAATTCGTT |
| IFN-β-F | AACAAGTGTCTCCTCCAAATTGC |
| IFN-β-R | CCACAGGAGCTTCTGACACTGA |
| MX1-F | CAGTCCAGCTCGGCAACAG |
| MX1-R | AAGGGATGTGGCTGGAGATG |
| H1N1-NP-F | GGGTGAAAATGGTCGAAGGACA |
| H1N1-NP-R | ATACACACAAGCAGGCAGGCAG |
| IFIT1-F | GCGCTGGGTATGCGATCTC |
| IFIT1-R | CAGCCTGCCTTAGGGGAAG |
| ISG15-F | TGGACAAATGCGACGAACCTC |
| ISG15-R | TCAGCCGTACCTCGTAGGTG |
| H13N2-NP-F | TGCCTGTGTGTATGGACTTGCTG |
| H13N2-NP-R | GGATTCTCATTGGGTCGGATTAG |
| RIG-I-F | TGGAGCAGGGAAGATGTTTG |
| RIG-I-R | GGTCCACACCTTCTCCATCA |

**Table S2. Sequences of shRNAs targeting FGF8 and TRIM16.**

| **Name** | **shRNA Sequence (5’-3’)** |
| --- | --- |
| **shFGF8-1** | CCGGCCTCTACATCTGCATGAACAACTCGAGTTGTTCATGCAGATGTAGAGGTTTTTT |
| **shFGF8-2** | CCGGAGCGTGAGGTCCACTTCATGACTCGAGTCATGAAGTGGACCTCACGCTTTTTTT |
| **shTRIM16-1** | CCGGGCCATTGTTCAGCGCAAATATCTCGAGATATTTGCGCTGAACAATGGCTTTTTT |
| **shTRIM16-2** | CCGGTACATAGGGCTGAAGGATAAACTCGAGTTTATCCTTCAGCCCTATGTATTTTTT |
